# Siddha *Varmathadaval* therapy in the management of stress-related insomnia: A case report

**DOI:** 10.1016/j.jaim.2026.101367

**Published:** 2026-10-13

**Authors:** Kanishka Sakthivel, Mary Princess Sulekha Stephen Rajaiah

**Affiliations:** a Sri Sairam Siddha Medical College and Research Centre, West Tambaram, Chennai, Tamil Nadu, India; b Sri Sairam Siddha Medical College and Research Centre, West Tambaram, Chennai, Tamil Nadu, India

**Keywords:** Siddha Medicine, *Varmathadaval* therapy, Insomnia, Stress-related sleep disorder, Traditional medicine, Non-pharmacological intervention

## Abstract

Insomnia affects 10-30% of adults globally, with limited non-pharmacological options. This single-case report documents Siddha *Varmathadaval* therapy for stress-related insomnia. A 55-year-old woman presented with four-month history of insomnia (difficulty initiating/maintaining sleep) and daytime fatigue. *Thookaminmai* (insomnia) of *Vatha*-*Pitham* origin per Siddha principles was diagnosed. Baseline assessments showed severe insomnia (Insomnia Severity Index [ISI] = 21/28, Pittsburgh Sleep Quality Index [PSQI] = 15/21, Athens Insomnia Scale [AIS] = 13/24, Perceived Stress Scale-10 [PSS-10] = 31/40). The patient underwent twenty-one consecutive daily *Varmathadaval* therapy sessions using *Sukku Thailam* (90-120 minutes each), administered by a trained Siddha physician. Post-therapy improvements included ISI = 8/28 (62% reduction), PSQI = 6/21 (60% reduction), AIS = 4/24 (69% reduction), and PSS-10 = 12/40 (61% reduction). Traditional Siddha parameters (*Manikadai Nool*, *Neikuri*) demonstrated progressive improvement correlating with clinical outcome. Sleep latency decreased (45 → 15 minutes) and total sleep time increased (4-5→6-7 hours). Benefits were sustained at 4-week (ISI = 9) and 12-week (ISI = 11) post-treatment follow-up, with mild stress-related variation. *Varmathadaval* therapy produced clinically significant, sustained improvements in stress-related insomnia. The therapy was safe and well-tolerated. Controlled trials with prospective stress assessment are warranted.

## 1 Introduction

### 1.1 Background

Insomnia affects 10-30% of adults globally [1]. Despite cognitive behavioral therapy for insomnia (CBT-I) being the gold standard non-pharmacological intervention [2], limited therapist access has increased interest in complementary therapies. Recent meta-analyses show acupressure significantly improves sleep quality (PSQI MD = −2.99, 95% CI: −4.95 to −1.04) [3], with acupuncture-herbal combinations demonstrating efficacy through stress hormone regulation and autonomic modulation [4].

### 1.2 Siddha medicine and Varmathadaval therapy

The Siddha medical system, originating in South India over 5000 years ago [5], conceptualizes insomnia as imbalances in three fundamental principles (Uyir Thathukkal): Vatham (kinetic energy/nervous function), Pitham (metabolic processes), and Kapham (structural stability). Varmathadaval therapy lacks systematic documentation using validated outcome measures.

### 1.3 Relevance to integrative medicine

This case exemplifies integrative medicine principles by combining traditional Siddha diagnostic methods (Naadi, Neikuri, Manikadai Nool) with contemporary validated assessment tools (ISI, PSQI, AIS, PSS-10). The integration provides a model for evaluating traditional medical systems within evidence-based healthcare frameworks, supporting therapeutic diversity in insomnia management.

## 2 Patient information

### 2.1 Demographics

A 55-year-old woman.

### 2.2 Chief complaint

Difficulty falling asleep and staying asleep for four months, with associated daytime fatigue.

### 2.3 Medical history

No significant medical, psychiatric, or sleep disorder history; no current medications or substance use.

### 2.4 Psychosocial history

Reports increased stress related to family responsibilities over the past six months, denying occupational stress or recent major life events.

## 3 Clinical findings

### 3.1 Physical examination

Vital signs: BP 120/78 mmHg, pulse 76/min, RR 16/min, temperature 98.2 °F.

General: Alert, cooperative, mildly fatigued. Systemic examination: Unremarkable.

### 3.2 Siddha diagnostic assessment

- Naadi (Pulse): Vatha-Pitham predominance
- Sparisam (Touch): Normal
- Naa (Tongue): Normal
- Niram (Complexion): Slightly pale
- Mozhi (Voice): Normal
- Vizhi (Eyes): Mild dark circles
- Malam/Moothiram: Normal
- Neikuri (Oil drop test): Vatha-Pitham pattern (streak elongates and tends to form circle)
- Manikadai Nool (Wrist circumference): 9.25 angulam (indication of insomnia and below normal range of 11 angulam, consistent with Vatha-Pitham derangement)

### 3.3 Routine laboratory investigations

Complete blood count, thyroid function, and fasting glucose were normal, ruling out organic causes.

### 3.4 Diagnosis

Thookaminmai (Insomnia) of Vatha-Pitham origin, corresponding to stress-related insomnia.

## 4 Timeline

See Fig. 1.

**Fig. 1:**
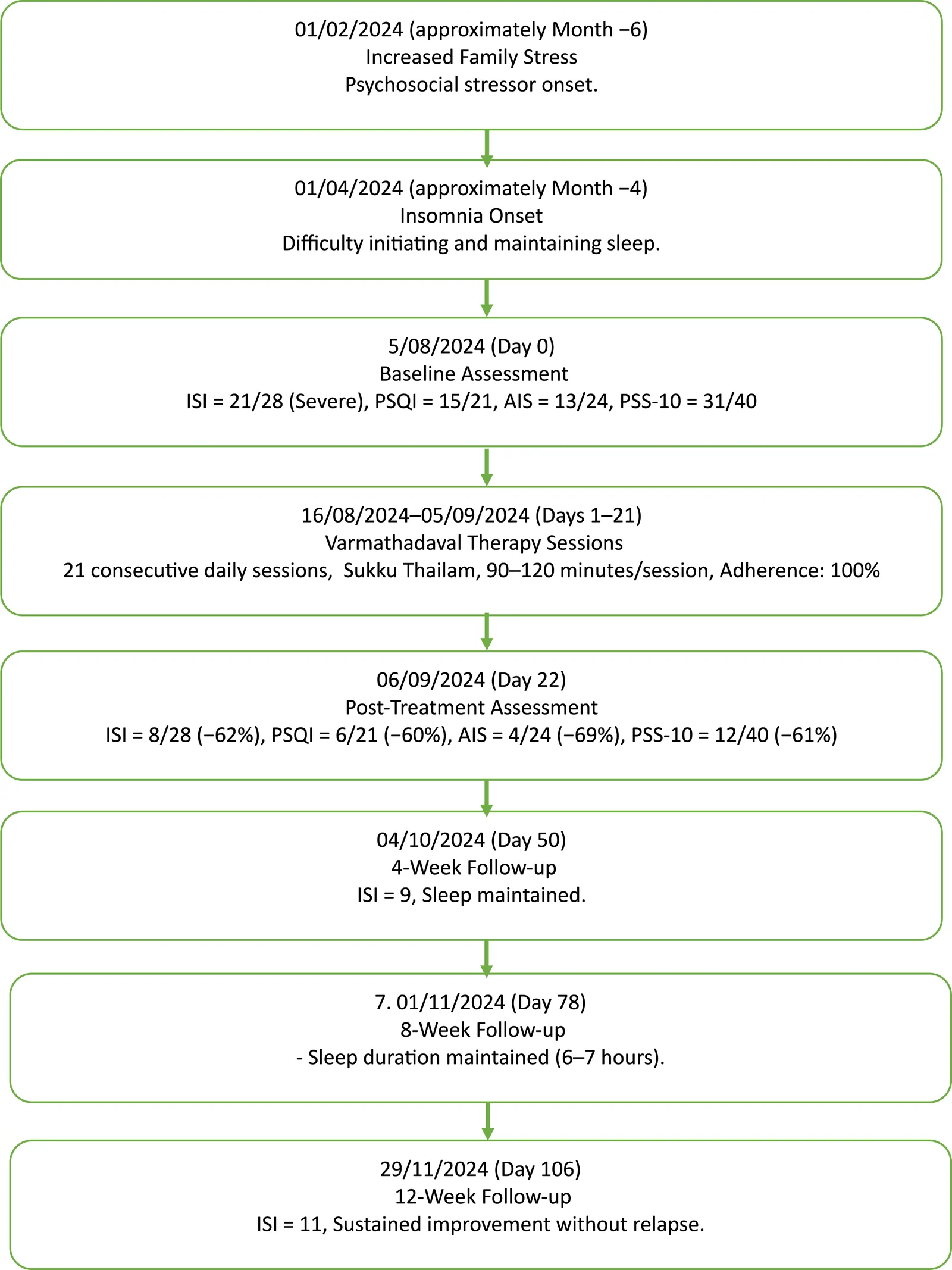
Clinical timeline of patient management.

## 5 Diagnostic assessment

Using both contemporary sleep assessment instruments and thorough Siddha diagnostic techniques, the diagnosis of Thookaminmai (insomnia) of Vatha-Pitham origin was established.

### 5.1 Siddha diagnostic rationale

Naadi examination revealed Vatha-Pitham predominance with clinical symptoms of mental restlessness, difficulty falling asleep, and frequent awakenings. Siddha principles recognize that elevated Pitham causes stress reactions while aggravated Vatham disrupts sleep-wake rhythm [6,7].

### 5.2 Sleep assessment tools

Three validated tools measured insomnia: Insomnia Severity Index (ISI) [8] baseline 21/28 (severe); Pittsburgh Sleep Quality Index (PSQI) [9] baseline 15/21 (poor); Athens Insomnia Scale (AIS) [10] baseline 13/24 (clinical insomnia). Sleep Diary [11] confirmed patterns. Perceived Stress Scale-10 (PSS-10) [12] baseline was 31/40.

## 6 Therapeutic intervention

### 6.1 Treatment protocol overview

The patient underwent 21 consecutive daily Varmathadaval therapy sessions, each lasting 90–120 minutes, administered by a single trained Siddha physician following a standardized 21-step craniofacial protocol using Sukku Thailam warmed to 37°C at 30–40 mL per session. The patient completed all 21 consecutive daily sessions without interruption (100% adherence).

### 6.2 Traditional Siddha diagnostic parameters

Manikadai Nool (wrist circumference) was measured using the patient's finger width as the unit (angulam) according to standardized Siddha methodology [6,13]. Neikuri (oil drop test) was performed using first-morning urine with gingelly oil according to classical Siddha principles [6,14]. Fig. 2 shows the photographic documentation was obtained at all three timepoints (baseline, Day 22, Week 4) with patient consent.

### 6.3 Medicated oil composition

Sukku Thailam contains gingerols and shogaols with documented anti-inflammatory and nervine properties [15]. While direct effects on insomnia lack extensive modern research, ginger's known anxiolytic properties may contribute synergistically to outcomes. Classical Siddha texts indicate Sukku Thailam's efficacy for Vatha-related nervous system disorders including sleep disturbances [7,[16], [17], [18]]. Transdermal absorption during extended sessions (90-120 minutes) may enhance mechanical manipulation effects, representing the traditional principle of combining medicated oil with Varma therapy.

### 6.4 Route of administration

Sukku Thailam was administered topically via manual massage of craniofacial Varma points. Oil warmed to 37°C; 30-40 mL per 90-120 minute session following 21-step protocol. No oral, rectal, nasal, or internal routes were used.

### 6.5 Anatomical correspondence of key Varma points

The targeted Varma points correspond to cranial nerve distributions (facial, trigeminal, vagus) and cerebrovascular structures as shown in Fig. 2, potentially modulating autonomic tone and cortical arousal networks implicated in sleep-wake regulation.

**Fig. 2:**
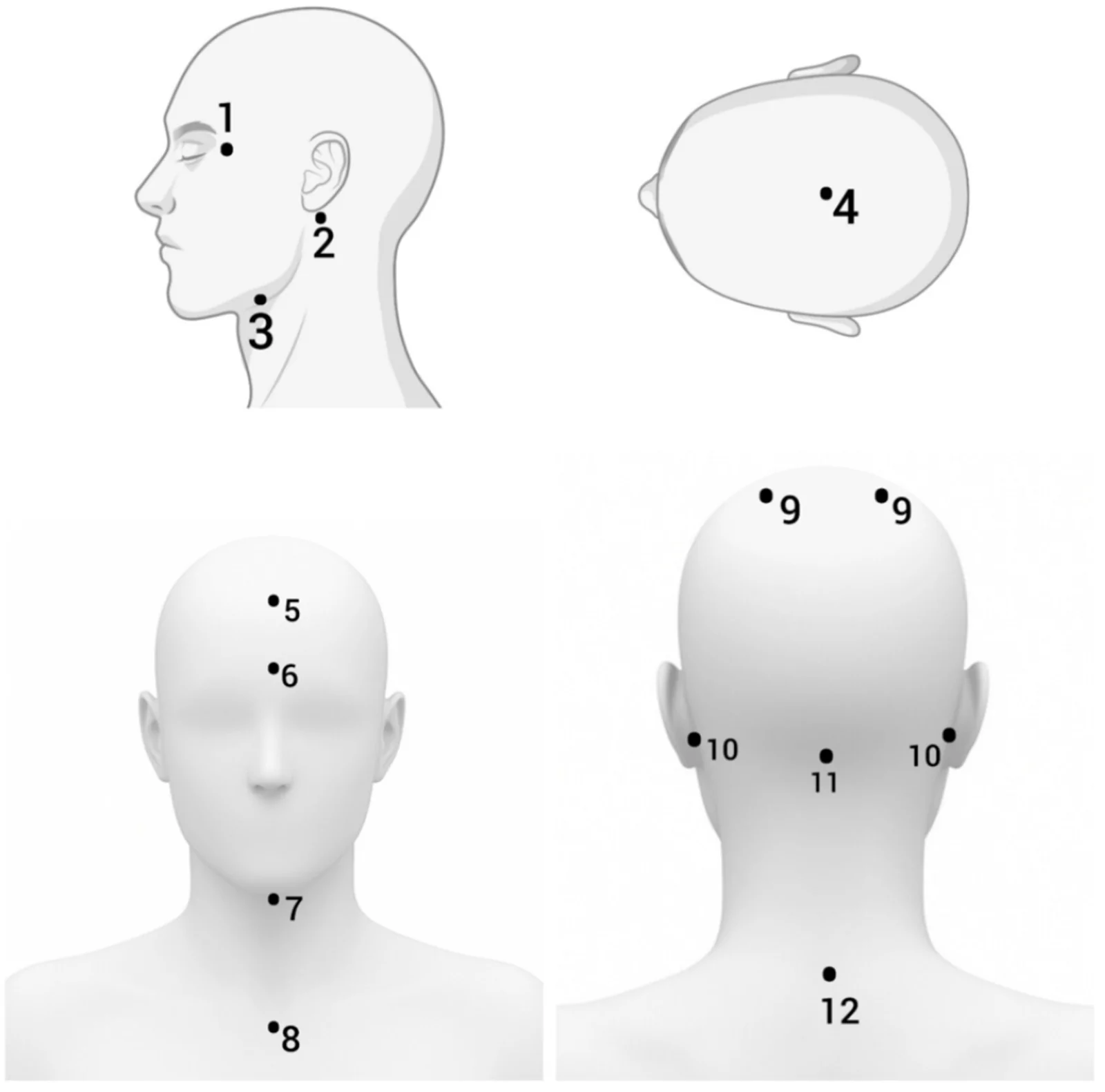
Anatomical locations of 12 principal Varma points (1. Natchathira kaalam, 2. Sevi kutri, 3. Urakka kaalam, 4. Uchi, 5. Moorka kalam, 6. Thilartha kaalam, 7. Ottu Varmam, 8. Thummi kaalam, 9. Thalai komberi, 10. Simil iruppu, 11. Pidari, 12. Valaimuduchu) targeted in therapy, overlaid on the craniofacial anatomy. Illustration created by authors based on classical Siddha texts [7,[16], [17], [18]].

### 6.6 Adjunctive measures and controls

Permitted: Normal activities, regular diet, sleep hygiene advice. Prohibited: No concurrent medications, supplements, alcohol, other therapies, or lifestyle changes during intervention.

### 6.7 Treatment monitoring

Daily monitoring included pre-session sleep inquiry, intra-session comfort assessment, and post-session adverse event surveillance.

### 6.8 Quality assurance

Standardization: Same therapist, oil batch, room, timing.

### 6.9 Statistical analysis

As a single-case study, formal inferential statistics were not applicable. Clinically meaningful change was assessed against established MCIDs: ISI ≥6 points, PSQI ≥3 points, AIS <6. Percentage changes and absolute differences were calculated. Sleep diary parameters are presented descriptively with pre-post comparisons.

## 7 Follow-up and outcomes

All outcome measures demonstrated clinically significant improvements exceeding established MCIDs, with traditional Siddha parameters corroborating clinical findings and benefits sustained through 12-week follow-up without adverse events.

### 7.1 Immediate post-treatment outcomes (Day 22)

A comprehensive reassessment revealed significant improvements in all parameters.

PSS-10 assessment showed stress reduction from 31/40 (baseline, Day 0) to 12/40 (post-treatment, Day 22), representing a 61.3% reduction. PSS-10 was not reassessed at subsequent follow-ups as insomnia remained the primary outcome. As shown in Table 1 (A), all three validated assessment tools demonstrated clinically significant improvements from baseline to post-therapy and sustained benefits.

**Table 1:** Pre- and post-Therapy.

PART A. Sleep Assessment Scores (Day 0 vs Day 22)						
S.no	Assessment Tool	Pre-Therapy (Day 0)	Post-Therapy (Day 22)	Absolute Change	Percentage Change	Clinical Significance
1.	Insomnia Severity Index (ISI)	21/28 (Severe)	8/28 (Subthreshold)	−13 points	−62%	Exceeded MCID of 6 points^a^
2.	Pittsburgh Sleep Quality Index (PSQI)	15/21 (poor)	6/21 (Good)	−9 points	−60%	Exceeded MCID of 3 points^b^
3.	Athens Insomnia Scale (AIS)	13/24 (clinical)	4/24 (Normal)	−9 points	−69%	Below clinical threshold of 6^c^
4.	PSS-10	31/40	12/40 points	−19	−61.3%	Sustained

PART B. Siddha Diagnostic Parameter (Day 0 vs Day 22 vs Week 4)					
Parameter	Baseline	Day 22	Week 4	Normal range	Interpretation
*Manikadai nool*	9.25 *angulam*	9.45 *angulam*	10.5 *angulam*	11 *angulam*	Progressive improvement, trending toward normal
*Neikuri*	*Vatha-pitham*	*Pitham*	*Kapham*	Balanced pattern	Humoral rebalancing

a Minimal clinically important difference (MCID) for ISI = 6 POINTS [8].

b MCID for PSQI = 3 POINTS [9].

c Clinical cut off for AIS = 6 POINTS [10]. (All three measures demonstrate clinically meaningful improvements exceeding established threshold values for clinical significance).

### 7.2 Traditional diagnostic parameters

Neikuri pattern changed from Vatha-Pitham (baseline) to Pitham (Day 22) to Kapham (Week 4), demonstrating progressive humoral rebalancing [6,14] (Fig. 3).

**Fig. 3:**
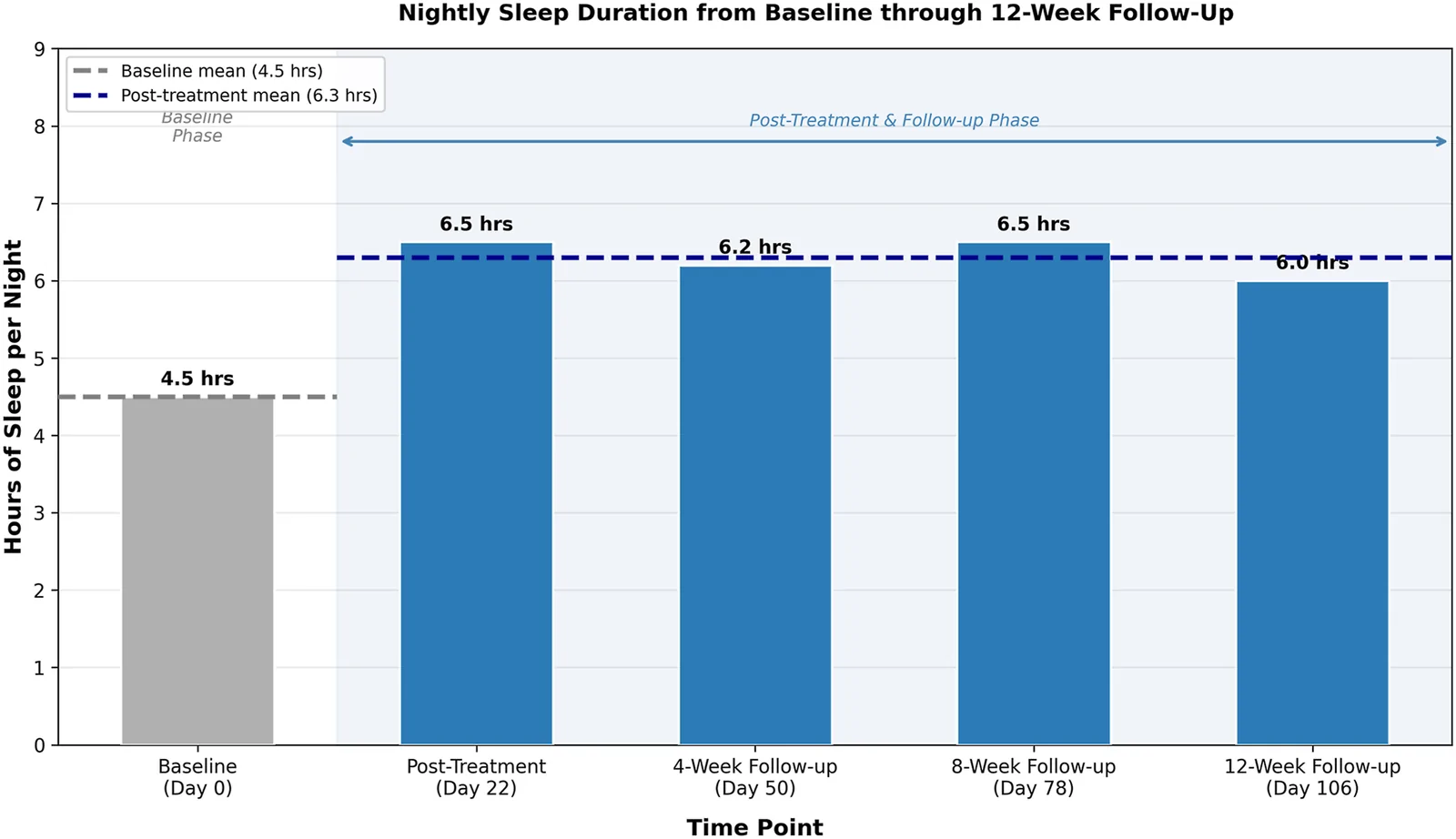
Nightly sleep duration (hours) from baseline assessment through 12-week post-treatment follow-up period. Grey shading indicates active treatment phase (Days 1-21). Data points represent total sleep time recorded in daily sleep diary. Despite normal night-to-night variability, sustained improvement in mean sleep duration is evident from baseline (4-5 hours) to post-treatment and follow-up periods (6-7 hours).

Manikadai Nool improved progressively from 9.25 angulam (baseline) to 9.45 (Day 22) to 10.5 (Week 4), approaching normal 11 angulam.

### 7.3 Secondary outcomes: sleep diary parameters

Sleep latency decreased (45 → 15 min), total sleep time increased (4.5 → 6.5 hrs), awakenings reduced (3.2 → 1.1/night), and sleep efficiency improved (65%→88%).

### 7.4 Tertiary outcomes: daytime functioning

Daytime functioning improved substantially across multiple domains.

### 7.5 Follow-up assessments

At 4 weeks post-treatment, ISI remained at 9. At 8 weeks, sleep duration of 6-7 hours was maintained. At 12 weeks, ISI was 11, indicating sustained improvement without relapse.

### 7.6 Safety and tolerability

Systematic adverse event monitoring revealed no skin reactions, anxiety, headaches, or musculoskeletal discomfort. Therapy was well-tolerated across all sessions.

## 8 Discussion

### 8.1 Clinical significance of findings

All three outcome measures exceeded established minimal clinically important differences [[8], [9], [10]] with categorical shifts from severe to subthreshold insomnia within 21 days despite four months of chronic symptoms. Siddha Varmathadaval therapy produced clinically significant improvements in severe stress-related insomnia within 21 consecutive daily sessions with sustained benefits through 12-week follow-up. Combining traditional Siddha diagnostic principles with validated modern instruments provides comprehensive evaluation for documenting traditional medicine outcomes.

### 8.2 Correlation of traditional and modern parameters

Traditional Siddha parameters corroborated improvements. Manikadai Nool increased 13.5% toward normal [6,13], paralleling sleep improvements (ISI 62%, PSQI 60%). Neikuri patterns demonstrated progressive humoral rebalancing [6,14].

### 8.3 Varma point mechanisms in insomnia management

The Varmathadaval protocol targets specific craniofacial Varma points with traditional indications for sleep disorders. According to classical Siddha texts [7,[16], [17], [18]], cranial points like Uchi (crown) and Pirai adangal (peri-auricular) regulate Vatha dosha, governing nervous system function and sleep-wake rhythms. The temporal-cervical points (Simil iruppu, Pidari) correspond anatomically to regions innervated by trigeminal and vagal nerves, potentially explaining stress reduction through autonomic modulation. Siddha principles [6,7] recognize that systematic stimulation of these points restores Vatha-Pitham balance, addressing both hyperarousal (elevated Pitham) and circadian disruption (aggravated Vatham) characteristic of stress-related insomnia. This traditional understanding aligns with modern concepts of manual therapy affecting autonomic nervous system balance through vagal and trigeminal pathways [19], suggesting plausible mechanistic pathways warranting controlled investigation. From a neurophysiological perspective, stimulation of craniofacial Varma points corresponding to trigeminal and vagal nerve territories may enhance parasympathetic activity and reduce sympathetic hyperarousal implicated in insomnia pathophysiology. Similar autonomic modulation mechanisms have been described for manual point stimulation therapies such as acupuncture [19].

### 8.4 Role of medicated oil in therapeutic outcomes

Sukku Thailam contains gingerols and shogaols - bioactive compounds with documented anti-inflammatory and neuroprotective properties [15] which may contribute to therapeutic outcomes through pharmacological mechanisms. While direct effects of ginger on insomnia lack extensive modern research documentation, its anxiolytic properties may contribute synergistically to outcomes. Classical Siddha texts describe Sukku Thailam as particularly effective for Vatha-related nervous system disorders including sleep disturbances [7,16]. The extended application duration (90-120 minutes) facilitates transdermal absorption, potentially providing pharmacological benefits beyond mechanical stimulation. This combination of medicated oil with specific Varma point manipulation represents a core Siddha therapeutic principle where the oil's properties enhance manual therapy effects, distinguishing this approach from simple massage therapy. Although direct clinical evidence of Sukku Thailam alone in insomnia is limited, ginger-derived bioactive compounds possess anxiolytic, anti-inflammatory, and neuroprotective effects relevant to stress and sleep regulation [15]. Therefore, the oil likely acts synergistically with Varma stimulation rather than as an independent hypnotic intervention.

### 8.5 Comparison with similar traditional interventions

Our findings align with meta-analytic evidence for manual therapies in insomnia. Acupressure studies report mean PSQI improvements of −2.99 points [3], while our protocol achieved −9 points, though the intensive multi-session approach may explain enhanced effect size. The sustained benefits through 12-week follow-up without additional treatment parallel CBT-I outcomes [2], suggesting that traditional manual therapies targeting specific anatomical points may produce durable adaptive changes. Unlike acupuncture requiring needle insertion, Varmathadaval's non-invasive approach may enhance patient acceptability. However, direct comparative trials are needed to establish relative efficacy, optimal treatment duration, and mechanisms distinguishing Varmathadaval from other manual therapies.

### 8.6 Sustained benefits

The 21-day duration approximates CBT-I duration [2], suggesting potential adaptive mechanisms.

### 8.7 Limitations

This single-case design limits causal inference; improvements may reflect natural variation or placebo. Outcomes were subjective without polysomnography. The patient had uncomplicated insomnia with cultural familiarity. Single therapist limits reproducibility. The protocol is based on classical Siddha texts [7,[16], [17], [18]] lacking peer-review but representing authentic primary sources with contemporary validation [20]. This provides preliminary hypothesis-generating data.

### 8.8 Future directions

Future randomised controlled trials with polysomnography and comparative effectiveness studies versus CBT-I [2] are warranted.

## 9 Patient perspective

The patient provided the following reflections (recorded Day 50, 4 weeks post-treatment):

“Before treatment, I struggled to sleep for months. After 2-3 sessions, I noticed easier sleep onset. By week two, I slept through the night for the first time in months. The therapy was relaxing. Weeks later, I’m still sleeping well with sustained energy. I would recommend this to others.”

## 10 Conclusion

This case documents clinically significant improvements in severe stress-related insomnia after 21 consecutive days of Siddha Varmathadaval treatment with substantial reductions in ISI (62%), PSQI (60%), AIS (69%), and PSS-10 (61%), sustained through 12-week follow-up. The therapy was safe and well-tolerated. These findings warrant replication in randomised controlled trials with objective sleep measures. Varmathadaval therapy may offer a non-pharmacological option for stress-related insomnia, particularly for patients seeking traditional medicine approaches.

## Patient consent

Written informed consent was obtained from the patient for participation, data collection, and publication of this case report including de-identified clinical photographs.

## Author contributions

Author 1: Conceptualization, clinical assessment, data collection, manuscript writing, and final approval.

Author 2: Varmathadaval therapy administration, clinical supervision, manuscript review, and final approval. Both authors meet all four ICMJE criteria for authorship.

## Data availability

The datasets generated and analysed during the current study are available from the corresponding author upon reasonable request, subject to patient privacy and institutional guidelines.

## Ethical approval

Throughout the study, patient autonomy, beneficence, non-maleficence, and confidentiality were upheld. For both treatment and publication, written informed consent was acquired.

## Declaration of Generative AI in scientific writing

During manuscript preparation, the authors used Claude 3.5 Sonnet (Anthropic, 2025) for grammar correction, language clarity, and formatting consistency. The AI tool was not used for data generation, analysis, interpretation or drawing clinical conclusions. All intellectual content, clinical observations, and scientific conclusions are entirely original work by the authors, who take full responsibility for the manuscript's accuracy and content.

## Funding statement

This research did not receive any specific grants from funding agencies in the public, commercial, or not-for-profit sectors. No financial support was received for this study.

## Conflict of Interest

The authors declare no conflicts of interest related to this study.
